# A 1D computer model of the arterial circulation in horses: An important resource for studying global interactions between heart and vessels under normal and pathological conditions

**DOI:** 10.1371/journal.pone.0221425

**Published:** 2019-08-21

**Authors:** Lisse Vera, Daimé Campos Arias, Sofie Muylle, Nikos Stergiopulos, Patrick Segers, Gunther van Loon

**Affiliations:** 1 Equine Cardioteam Ghent University, Dept. of Large Animal Internal Medicine, Faculty of Veterinary Medicine, Ghent University, Ghent, Belgium; 2 IBiTech-bioMMeda, Ghent University, Ghent, Belgium; 3 Biomechanics and Biomaterials Research Group, CUJAE, Havana, Cuba; 4 Dept. of Morphology, Faculty of Veterinary Medicine, Ghent University, Ghent, Belgium; 5 Laboratory of Hemodynamics and Cardiovascular Technology, EPFL, Lausanne, Switzerland; Boston University, UNITED STATES

## Abstract

Arterial rupture in horses has been observed during exercise, after phenylephrine administration or during parturition (uterine artery). In human pathophysiological research, the use of computer models for studying arterial hemodynamics and understanding normal and abnormal characteristics of arterial pressure and flow waveforms is very common. The objective of this research was to develop a computer model of the equine arterial circulation, in order to study local intra-arterial pressures and flow dynamics in horses. Morphologically, large differences exist between human and equine aortic arch and arterial branching patterns. Development of the present model was based on post-mortem obtained anatomical data of the arterial tree (arterial lengths, diameters and branching angles); *in vivo* collected ultrasonographic flow profiles from the common carotid artery, external iliac artery, median artery and aorta; and invasively collected pressure curves from carotid artery and aorta. These data were used as input for a previously validated (in humans) 1D arterial network model. Data on terminal resistance and arterial compliance parameters were tuned to equine physiology. Given the large arterial diameters, Womersley theory was used to compute friction coefficients, and the input into the arterial system was provided via a scaled time-varying elastance model of the left heart. Outcomes showed plausible predictions of pressure and flow waveforms throughout the considered arterial tree. Simulated flow waveform morphology was in line with measured flow profiles. Consideration of gravity further improved model based predicted waveforms. Derived flow waveform patterns could be explained using wave power analysis. The model offers possibilities as a research tool to predict changes in flow profiles and local pressures as a result of strenuous exercise or altered arterial wall properties related to age, breed or gender.

## Introduction

A wide range of one dimensional (1D) computer models of the human arterial circulation is available. Such models allow the computation of pressure and flow waveforms throughout the whole arterial network, and hence allow researchers to study the normal and abnormal physiology of the cardiovascular system, without the need of *in vivo* measurements [1–7]. 1D models are well-balanced between complexity and computation costs, making them relevant for many (bio)medical applications. Due to their capability of involving extensive arterial segments, 1D models can provide useful information about characteristics of blood flow at the level of individual branches or even in patient-specific situations [8, 9]. These models can also be used as a non-invasive diagnostic tool, helping physicians to understand observed changes in routine clinical blood pressure measurements and their possible physiological origin and to predict surgical operation results [9–11].

Due to technical limitations, difficult arterial accessibility, and ethical concerns, in-depth pathophysiological research of the equine arterial tree remains challenging and could be facilitated by the application of a model. Because of large differences between human and equine arteries regarding dimensions and branching patterns, especially of the aortic arch, a horse specific 1D model of the arterial circulation is needed.

The aim of this study was therefore to develop a 1D computer model of the equine arterial circulation. Providing reference data on equine arterial hemodynamics and physiology, this model might contribute to a better understanding of some clinical findings, such as the origin of the more oscillatory flow patterns in horses, the higher prevalence of aortic rupture in Friesians compared to Warmblood horses [12, 13], the occurrence of sudden death during exercise due to arterial rupture [14–17], the higher chance on uterine artery rupture in older mares [18, 19], or the higher chance on arterial rupture after phenylephrine administration in older horses [20]. In order to develop a reliable model, several anatomical data of the main equine arterial tree were collected *ex vivo* and combined both with *in vivo* invasive blood pressure measurements and non-invasively determined ultrasonographic flow profiles.

## Materials and methods

All procedures were approved by the Ethical Committee of the Faculty of Veterinary Medicine, Ghent University (EC 2016/104). Five warmblood horses were investigated, mean age 18 ± 3 years and mean body weight 648 ± 47 kg. All horses were scheduled for euthanasia because of non-cardiovascular reasons. One horse was privately owned (informed owner consent was obtained), the remaining four horses were experimental horses owned by the Faculty of Veterinary Medicine, Ghent University. For euthanasia the following protocol was applied: premedication with detomidine 0.02mg/kg; induction with a combination of ketamine 2.2mg/kg I.V. and midazolam 0.04mg/kg I.V.; and finally euthanasia with 6 ml/kg T61 (Intervet International GmbH, Unterschleissheim, Germany), containing 24mg/kg embutramide, 6 mg/kg mebezoniumjodide and 0,6 mg/kg tetracaïnehydrochloride.

### In vivo measurements

#### Ultrasound

Ultrasound imaging was performed (Vivid IQ, GE Healthcare) on all 5 standing, non-sedated horses. Different regions along the arterial tree were examined: the aorta from a left and right parasternal position, the right common carotid artery 15 cm cranial to the thoracic inlet, the right external iliac artery from the inguinal region and the right median artery just proximal to the carpus on the medial side of the leg. 2D B-mode images were collected, using a 9 MHz linear transducer (9L-RS, GE Healthcare) for the common carotid and the median artery, a 6 MHz phased array probe (6S-RS,GE Healthcare) for the external iliac artery and a 5 MHz phased array probe (M5Sc-RS, GE Healthcare) for the aorta. Mean values were obtained from 3 consecutive cardiac cycles at a heart rate between 35–45 beats per minute. Measurements were performed off-line (Echopac version 201, GE Healthcare). Diastolic diameters were measured from a transverse image for the carotid and external iliac artery, and from a longitudinal image for the aorta and the median artery. Pulsed wave Doppler images were collected at every location, using a 6 MHz phased array probe (6S-RS,GE Healthcare) for the common carotid and the external iliac artery, a 5 MHz phased array probe (M5Sc-RS GE Healthcare) for the aorta, and a 9 MHz linear probe (9L-RS,GE Healthcare) for the median artery. Angle correction was set at 45° for every image at all locations. Using this fixed angle correction, images were optimised to align with the flow direction.

#### Invasive blood pressure

In all 5 horses, the blood pressure at the level of the common carotid artery was measured invasively in the standing, awake animal. The right common carotid artery was punctured aseptically under ultrasound guidance (Vivid IQ, GE Healthcare; 9L-RS, GE Healthcare), using an 18 gauche 90mm needle (Terumo spinal needle, Terumo) placed in the middle of the lumen and kept in place for at least 20 consecutive heart cycles. The needle was connected with a fluid filled pressure transducer (MLT0699 Disposable BP Transducer, ADInstruments) interfacing with a digital acquisition station (PowerLab 8, ADInstruments), blood pressure curves were recorded for offline analysis (LabChart, ADInstruments). For each horse the systolic, diastolic and mean arterial pressure was calculated automatically as the mean of 20 consecutive heart beats (heart rate between 35–45 bpm).

In one horse, scheduled for euthanasia, invasive blood pressure measurements over the whole length of the thoracic and abdominal aorta were performed under general anaesthesia (pre-medication: detomidine 0.02mg/kg; induction: combination of ketamine 2.2mg/kg I.V. and midazolam 0.04mg/kg I.V.) with the horse in the dorsal recumbent position. After chirurgical exposure of the right common carotid artery, a 72cm steerable 8.5Fr sheath (Zurpaz, Boston Scientific) was introduced using the Seldinger technique. Under transthoracic ultrasound guidance (Vivid IQ, GE Healthcare; M5Sc-RS, GE Healthcare) the sheath was introduced retrogradely through the brachiocephalic trunk, into the ascending part of the aorta. Once in place, a custom-made pressure tip catheter (Gaeltec) was introduced and advanced to the most caudal end of the aorta under transrectal ultrasound guidance. Blood pressures were recorded (PowerLab 8, ADInstruments) at the most caudal site and subsequently at every 10 cm during step-wise pulling back of the catheter until the ascending part of the aorta was reached. An ECG was recorded simultaneously. At each location, an ensemble-averaged waveform was constructed from at least 5 cardiac cycles. The ensemble-average was aligned in time relative to the peak of the R-wave of the ECG. Pulse wave velocity was calculated from the relation between inter-measurement distance and time delay. The time delay was calculated from delays in the peak of the 2^nd^ derivative of pressure, assumed to represent the foot of the pressure wave. After the procedure the horse was euthanized while still under general anaesthesia with 6 ml/kg bodyweight T61 (Intervet International GmbH, Unterschleissheim, Germany) containing 24mg/kg bodyweight embutramide, 6 mg/kg bodyweight mebezoniumjodide and 0,6 mg/kg bodyweigth tetracaïnehydrochloride.

### Ex vivo measurements

Necropsy of 4 out of 5 horses was performed within 12 hours after euthanasia. A dissection was completed on the aorta and the most important (left sided) side branches and morphometric data (length, diameter and branching angle) were recorded. Arterial length was measured using a tape measure and diameters were measured in the middle of each segment by introducing custom-made iron rods of different diameters into the explored arterial lumen. Subsequently, post-mortem diameters were scaled to the *in vivo* diameters, using the *in vivo* ultrasound measurements of common carotid artery, external iliac artery and median artery as a reference. Angles of the different arterial segments in the 1-dimensional plane were measured using anatomical images of the equine arterial tree [21]. Lengths of terminal segments were only measured in one horse. Right sided circulation was assumed to be the same as the left sided arterial circulation, except for the right sided subclavian circulation (as the branching pattern is different), which was measured separately in one of the horses. All investigated arterial branches and their corresponding average length and diameter are displayed in Table 1. Note that in the mathematical model vessel tapering will be included, meaning that average segment diameters will be adjusted to minimize forward reflections (see chapter ‘Distal boundary conditions and bifurcations’).

**Table 1:** Anatomical data of the equine arterial tree.

Artery	Arterial Segment Number	Angle in the 1D plane (degrees)	Mean Length (mm)	SD	Mean diameter (mm)	SD	Tapering		Distensibility 10^−3^ 1/mmHg
							Proximal lumen diameter (mm)	Distal lumen diameter (mm)	
**Aorta ascendens 1**	1	90	18	9	68	6	68.18	67.39	6.85
**A coronaria sinistra**	2	180	20	/	19	6	23.638	12.10	3.17
**A coronaria dextra**	3	0	20	/	19	6	23.638	12.10	3.17
**Aorta ascendens 2**	4	90	64	9	67	6	67.22	66.82	6.80
**Truncus brachiocephalicus 1**	5	160	38	26	39	9	38.882	38.88	4.90
**A subclavia sinistra 1**	6	155	39	24	26	11	25.332	25.33	3.79
**Truncus costocervicalis sinister**	7	90	10	/	11	5	12.102	10.50	2.34
**A subclavia sinistra 2**	8	155	19	13	24	8	24.11	24.11	3.68
**A cervicalis profunda sinistra**	9	115	70	/	3	1	2.82	2.82	0.87
**A subclavia sinistra 3**	10	155	14	8	24	8	24.11	24.11	3.68
**A vertebralis sinistra**	11	135	95	/	12	5	14.17	9.98	2.45
**A subclavia sinistra 4**	12	240	40	6	23	6	22.89	22.89	3.57
**A thoracica interna sinistra**	13	280	137	/	12	4	12.11	12.11	2.44
**A subclavia sinistra 5**	14	240	7	8	23	6	22.89	22.89	3.57
**A cervicalis superficialis sinistra**	15	180	80	/	8	2	10.31	4.79	1.90
**A axillaris sinistra 1**	16	310	62	25	21	6	21.50	20.37	3.38
**A suprascapularis sinistra**	17	70	15	/	6	0	7.68	3.52	1.59
**A axillaris sinistra 2**	18	310	58	45	20	8	19.90	19.90	3.28
**A subscapularis sinistra**	19	10	10	/	17	7	16.80	16.80	2.96
**A axillaris sinistra 3**	20	310	23	18	18	5	18.02	18.02	3.09
**A circumflexa humeri cranialis sinistra**	21	230	30	/	5	2	6.11	2.79	1.39
**A axillaris sinistra 4**	22	270	70	28	17	3	17.05	17.05	2.99
**A profunda brachii sinistra**	23	325	5	/	11	5	11.281	9.60	2.23
**A axillaris sinistra 5**	24	270	82	11	14	1	15.35	12.79	2.67
**A collateralis ulnaris sinistra**	25	320	5	/	6	2	8.60	4.92	1.75
**A mediana sinistra**	26	270	145	/	7	3	10.01	5.30	1.90
**Truncus brachiocephalicus 2**	27	160	50	/	39	/	38.88	38.88	4.90
**Truncus costocervicalis dexter**	28	90	10	/	11	6	14.38	7.07	2.34
**Truncus brachiocephalicus 3**	29	160	10	/	39	/	38.88	38.88	4.90
**A cervicalis profunda dextra**	30	115	70	/	3	2	2.81	2.81	0.87
**Trunucus brachiocephalicus 4**	31	160	10	/	39	/	38.88	38.88	4.90
**A vertebralis dextra**	32	135	95	/	12	5	16.95	7.12	2.54
**Truncus brachiocephalicus 5**	33	160	20	/	26	/	26.96	25.00	4.24
**A subclavia dextra 1**	34	240	30	/	26	11	25.00	25.00	3.76
**A thoracica interna dextra**	35	280	137	/	11	5	12.12	12.10	2.44
**A cervicalis superficialis dextra**	36	180	80	/	3	1	8.07	8.02	1.90
**A axillaris dextra 1**	37	310	62	25	21	6	20.95	20.94	3.38
**A suprascapularis**	38	70	15	/	6	0	6.43	5.47	1.59
**A axillaris dextra 2**	39	310	58	45	20	8	19.90	19.90	3.28
**A subscapularis dextra**	40	10	10	/	17	7	16.80	16.80	2.96
**A axillaris dextra 3**	41	310	23	18	18	5	18.02	18.02	3.09
**A circumflexa humeri cranialis dextra**	42	230	30	/	5	2	6.115	2.79	1.39
**A axillaris dextra 4**	43	270	70	28	17	3	17.05	17.05	2.99
**A profunda brachii dextra**	44	325	5	/	11	5	10.47	10.47	2.23
**A axillaris dextra 5**	45	270	82	11	14	1	17.05	10.42	2.67
**A collateralis ulnaris dextra**	46	320	5	/	6	2	8.60	4.92	1.75
**A mediana dextra**	47	270	145	/	7	3	10.00	5.30	1.90
**Truncus bicaroticus**	48	165	78	91	22	19	22.06	21.26	3.45
**A carotis communis sinistra**	49	110	710	59	12	1	13.45	10.35	2.42
**A carotis interna sinistra**	50	90	120	/	4	1	4.43	3.53	1.25
**A occipitalis sinistra**	51	60	45	/	5	2	4.94	4.13	1.35
**A carotis externa sinistra 1**	52	130	65	41	10	3	10.04	10.04	2.18
**Truncus linguofacialis sinister**	53	180	80	/	6	3	6.54	5.50	1.60
**A carotis externa sinistra 2**	54	90	41	11	9	2	9.22	8.83	2.04
**Ramus massetericus sinister**	55	255	20	/	2	1	2.51	1.21	0.82
**A carotis externa sinistra 3**	56	90	13	3	9	2	8.56	8.55	1.98
**A auricularis caudalis sinistra**	57	55	10	/	3	2	3.953	1.74	1.07
**A carotis externa sinistra 4**	58	90	23	3	7	3	8.56	5.15	1.76
**A temporalis superficialis sinistra**	59	60	20	/	2	2	3.44	2.48	1.05
**A carotis externa sinistra 5**	58b*	90	5		5		5.15	5.15	1.46
**A carotis communis dextra**	60	110	710	59	12	1	13.45	10.35	2.42
**A carotis interna dextra**	61	90	120	/	4	1	4.43	3.53	1.25
**A occipitalis dextra**	62	60	45	/	5	2	4.94	4.13	1.35
**A carotis externa dextra 1**	63	130	65	41	10	3	10.04	10.04	2.18
**Truncus linguofacialis dexter**	64	180	80	/	6	3	6.54	5.50	1.60
**A carotis externa dextra 2**	65	90	41	11	9	2	9.22	8.83	2.04
**Ramus massetericus dexter**	66	255	20	/	2	1	2.51	1.21	0.82
**A carotis externa dextra 3**	67	90	13	3	9	2	8.56	8.55	1.98
**A aurocularis caudalis dextra**	68	55	10	/	3	2	3.95	1.74	1.07
**A carotis externa dextra 4**	69	90	23	3	7	3	8.56	5.15	1.76
**A temporalis superficialis dextra**	70	60	20	/	2	2	3.44	2.48	1.05
**A carotis externa dextra 5**	69b*	90	5		5		5.15	5.15	1.46
**Arcus aortae (lig. Art Botalli)**	71	45	77	26	54	10	58.07	49.53	5.97
**Aorta descendens 1**	72	45	105	5	45	11	48.21	40.62	5.32
**A broncho-oesophagea**	73	270	66	/	11	2	13.71	6.31	2.26
**Aorta descendens 2**	74	0	486	69	36	1	38.44	32.28	4.64
**A coeliaca**	75	270	20	/	15	3	19.31	9.24	2.78
**Aorta descendens 3**	76	0	48	26	29	6	28.70	28.70	4.09
**A mesenterica cranialis**	77	270	20	/	11	7	13.48	7.56	2.29
**Aorta descendens 4**	78	0	30	35	29	10	28.70	28.70	4.09
**A renalis dextra**	79	270	30	/	11	2	13.26	7.21	2.26
**Aorta descendens 5**	80	0	20	19	29	5	28.70	28.70	4.09
**A renalis sinistra**	81	270	30	/	11	2	13.26	7.21	2.26
**Aorta descendens 6**	82	0	104	60	29	6	28.70	28.70	4.09
**A ovarica sinistra**	83	270	300	/	3	1	2.76	2.76	1.00
**A ovarica dextra**	84	270	300	/	3	1	2.76	2.76	1.00
**Aorta descendens 7**	85	0	40	27	29	6	28.70	28.70	4.09
**A mesenterica caudalis**	86	270	10	/	8	3	9.78	4.63	1.85
**Aorta descendens 8**	87	0	63	12	29	6	28.70	28.70	4.09
**A iliaca interna sinistra**	88	330	30	10	18	6	20.19	15.12	0.92
**A iliaca externa sinistra 1**	89	315	30	/	17	2	16.93	16.24	0.88
**A circumflexa iliumprofunda sinistra**	90	240	160	/	6	2	7.58	3.66	0.48
**A iliaca externa sinistra 2**	91	280	29	19	16	3	15.99	15.54	0.86
**A uterina sinistra**	92	260	200	/	3	2	3.19	3.19	0.33
**A iliaca externa sinistra 3**	93	280	230	54	15	3	15.30	15.30	0.84
**A profunda femoris sinistra**	94	335	30	/	8	2	7.93	7.23	0.55
**A femoralis sinistra 1**	95	280	51	14	15	3	15.26	14.27	0.82
**A circumflexa femoris lateralis sinistra**	96	240	30	/	6	3	7.69	3.62	0.48
**A femoralis sinistra 2**	97	280	180	56	11	1	10.78	10.78	0.68
**A saphena sinistra**	98	285	625	/	3	3	2.59	2.59	0.29
**A femoralis sinistra 3**	99	280	25	26	10	1	10.78	9.71	0.66
**A genus descendens sinistra**	100	260	25	/	3	1	4.46	3.47	0.38
**A femoralis sinistra 4**	99b*	280	5		10		9.71	9.71	0.64
**A iliaca interna dextra**	101	330	30	10	18	6	20.19	15.12	0.92
**A iliaca externa dextra 1**	102	315	30	/	17	2	16.98	16.19	0.88
**A uterina dextra**	103	260	200	/	6	2	3.19	3.19	0.33
**A iliaca externa dextra 2**	104	280	29	19	16	3	15.97	15.56	0.86
**A circumflexa iliumprofunda dextra**	105	240	160	/	3	2	7.52	3.77	0.48
**A iliaca externa dextra 3**	106	280	230	54	15	3	15.30	15.30	0.84
**A profunda femoris dextra**	107	335	30	/	8	2	7.93	7.23	0.55
**A femoralis dextra 1**	108	280	51	14	15	3	15.26	14.27	0.82
**A circumflexa femoris lateralis dextra**	109	240	30	/	6	3	7.69	3.62	0.48
**A femoralis dextra 2**	110	280	180	56	11	1	10.78	10.78	0.68
**A saphena dextra**	111	285	625	/	3	3	2.59	2.59	0.29
**A femoralis dextra 3**	112	280	25	26	10	1	10.78	9.71	0.66
**A genus descendens dextra**	113	260	25	/	3	1	4.46	3.47	0.38
**A femoralis dextra 4**	112b*	280	5		1		9.71	9.71	0.64

SD: standard deviation

/: no SD could be obtained, because the measurement was only performed in one horse

* An additional terminal segment, which was not measured on necropsy, with an artificial length of 5 cm was implemented in the model.

### Mathematical model

The mathematical model is based on a previously published and validated 1D arterial network model in humans (22, 23). Main differences between the human and the adapted equine model are described below. The system of nonlinear equations is solved using in-house MatLab code. For the solution an implicit finite difference scheme was chosen, with second order of accuracy for the temporal and spatial domains. Forward, central and backward difference approximations of the spatial derivative were used for the proximal, middle and distal nodes in each segment, respectively. The arterial tree was initialized with a pressure of 100 mmHg and a flow of 1ml/s. The solution was found over 8 cardiac cycles yielding pressure and flow waveforms over the entire arterial tree. For more details on the modelling aspects and the mathematical equations, we refer the interested reader to Ref. (22).

#### Governing equations

**Fig 1:**
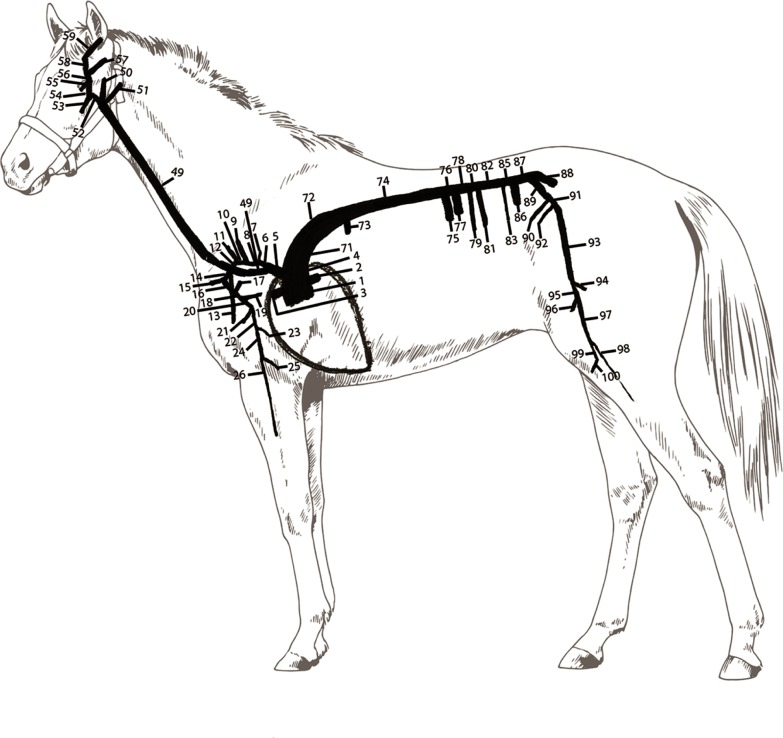
Schematic representation of the left sided arterial tree of the horse. Numbers agree with the numbers displayed in Table 1.

The main branches of the equine arterial tree were divided into 117 interconnected straight cylindrical arterial segments and inserted in the mathematical model (Fig 1, Table 1). The integrated forms of the continuity and momentum equations of the Navier-Stokes equations were solved in each of these segments for pressure (P), flow (Q) and cross-sectional area (A),

$$\frac{\partial A}{\partial t}+\frac{\partial Q}{\partial x}=0$$ (1)

$$\frac{\partial Q}{\partial t}+\frac{\partial }{\partial x}(\int_{A}u^{2}dA)=-\frac{A}{\rho }\frac{\partial P}{\partial x}-{2\pi R\frac{\mu }{\rho }\frac{\partial u}{\partial r}|}_{r=R}+Ag\;\cos\;\theta$$ (2)

where *x* and *t* are the spatial and temporal variables, *u* is the longitudinal velocity component, and *R* is the lumen radius. Given that measurements in animals, used as input into the model, were acquired in standing, awake animals, as well as in anesthetized and supine animals, we expect effects of gravity to be important given the height of the animal. We therefore accounted for the effects of gravity including the body forces term in the momentum equation, with *g* = 9.81 m/s^2^, the gravitational acceleration constant, and *θ*, the projection angle on the vertical axis. Results will be further reported for both situations, considering the effects of gravity and neglecting gravitational body forces. Blood was assumed to be an incompressible Newtonian fluid with density ρ = 1050 kg/m^3^ and dynamic viscosity μ = 0.004 Pa∙s. Given the large arterial diameters in horses, the Witzig-Womersley correction (see section ‘Velocity profile’) was used to approximate the convective acceleration term ($\frac{\partial }{\partial x}(\int_{A}u^{2}dA)$) and the wall friction term (${\tau =\mu \frac{\partial u}{\partial r}|}_{r=R}$), both present in the momentum equation.

#### Modelling of the arterial wall

A constitutive equation is needed to account for the elastic properties of the arterial wall, relating the dependency in intra-arterial pressure with the cross-sectional area. The nonlinear elastic behaviour of the arterial wall is assumed with an expression of area compliance (C_A_) [22], as the product of a location-dependent function, $C_{d}(\bar{d},P_{ref})$, and a pressure-dependent function, C_p_(P).

$$C_{A}(\bar{d},P)=\underset{C_{d}(\bar{d},P_{ref})}{\underbrace{\frac{A}{\rho ∙{PWV}^{2}(\bar{d},P_{ref})}}}∙\underset{C_{p}(P)}{\underbrace{\left[a_{1}+\frac{b_{1}}{1+{\left[\frac{P-P_{maxC}}{P_{width}}\right]}^{2}}\right]}}$$ (3)

The function $C_{d}(\bar{d},P_{ref})$ gives the compliance for a given local mean arterial lumen diameter, $\bar{d}$, at a given reference pressure value, P_ref_ = 100 mmHg. Reymond et al. [22] fitted an empirical inverse power curve for PWV as a function of $\bar{d}$, from human data reported in the literature.

$$PWV(\bar{d})\approx \frac{a_{2}}{{\bar{d}}^{b_{2}}}$$ (4)

To the best of our knowledge, measurements of the pressure and diameter dependency of the compliance in horses are lacking in literature but it can be assumed that the intrinsic building blocks (elastin, collagen, smooth muscle cells, proteoglycans) and their organization, and hence the overall mechanical behaviour, is similar as in humans. Initially, following Reymond et al. [22], we used the fitted values on humans for the pressure dependency of the compliance. However, these parameters were subsequently adapted to obtain values of pulse pressure close to the value reported by Boegli et al. [23] for healthy horses, resulting in a_1_ = 0.76, b_1_ = 5, P_maxC_ = 10 mmHg and P_width_ = 21 mmHg. We also applied the fit obtained from human data to describe the relation between local diameter and compliance (for a_2_ = 13.3 and b_2_ = 0.3). Nonetheless, we did tune the arterial distensibilities to the equine physiology, by multiplying the distensibilities of all vessels of the 1D model by a common factor with value 0.75. That factor was determined such that in resting conditions, we obtained a value of PWV close to the PWV computed from invasive blood pressure measurements over the abdominal aorta of an anesthetized horse; keeping in mind that differences may arise between an anesthetized and a conscious animal.

#### Velocity profile

The Witzig-Womersley theory describes the effect of flow pulsatility and inertia on the velocity profile. This oscillatory flow theory was needed to calculate the terms of convective acceleration and wall shear stress in the momentum equation derived from the Navier-Stokes equations, given that both terms depend on the instantaneous velocity profile. The Witzig-Womersley theory requires the knowledge of the local flow profile across the arterial lumen over the entire heart cycle, which is a priori unknown in the 1D model. This difficulty was overcome assuming that the solution is periodic; the flow waveform from the previous heart cycle was used to calculate the velocity profile and the wall shear stress using the formulations:

$$u(r,t)=\frac{2}{\pi R^{2}}\left(1-\frac{r^{2}}{R^{2}}\right)Q_{1}+\sum_{n=2}\mathrm{Real}\left\{\frac{Q_{n}}{\pi R^{2}}\frac{1-\frac{J_{0}(\alpha i^{3/2}r/R)}{J_{0}(\alpha i^{3/2})}}{1-\frac{2J_{1}(\alpha i^{3/2})}{\alpha i^{3/2}J_{0}(\alpha i^{3/2})}}e^{i\omega t}\right\}$$ (5)

$$\tau (t)=-\frac{4\mu }{\pi R^{3}}Q_{1}+\sum_{n=2}\mathrm{Real}\left\{\frac{\mu }{\pi R^{3}}Q_{n}\alpha i^{3/2}\frac{\frac{J_{1}(\alpha i^{3/2})}{J_{0}(\alpha i^{3/2})}}{1-\frac{2J_{1}(\alpha i^{3/2})}{\alpha i^{3/2}J_{0}(\alpha i^{3/2})}}e^{i\omega t}\right\}$$ (6)

The velocity profile (u) and the wall friction term (*τ*) are calculated as a Fourier series with harmonics (*n*) and depend on the harmonic-specific Womersley’s number $\alpha =R\sqrt{\rho 2\pi f/\mu }$, where R is the artery radius and f the frequency, *r*/*R* is the relative radial position, *Q*_*n*_ is the *n*^th^ harmonic of the flow pulse, and *J*_0_ and *J*_1_ are the Bessel functions of first kind of order 0 and 1, respectively. The oscillatory flow theory is taken into account for vessels with α>3 (75% of the total number of arterial segments), where the wall friction and convective acceleration are related as independent terms in the 1D momentum equation; the solution described by Stergiopulos et al.[3] is considered otherwise. The solution is found solving in time a number of repeating cycles until convergence.

#### Distal boundary conditions and bifurcations

For the terminal nodes a 3-element Windkessel model was used, to account for the cumulative effect of all distal vessels beyond the terminal sites [3, 22]. The equation takes the form:

$$\frac{\partial Q}{\partial t}=\frac{1}{R_{1}}\frac{\partial P}{\partial t}+\frac{P}{R_{1}R_{2}C_{T}}-\left(1+\frac{R_{1}}{R_{2}}\right)\frac{Q}{R_{1}C_{T}}$$ (7)

where R_1_ is the proximal resistance, R_2_ is the distal resistance, and C_T_ is the terminal compliance. Total peripheral resistances R_T_ = R_1_+R_2_, were estimated taking into account both the distribution of flow described in the literature [24] and our own measurements of flow at specific locations from ultrasound data. A total resistance parallel combination of about 0.14 mmHg*s/ml was assumed. The values of R_1_ were estimated assuming minimal reflection at high frequencies, with the condition R_1_ = Z_c_, where Z_c_ = ρ∙PWV/A is the characteristic impedance of the terminal segments. The values of distal resistance were then calculated as R_2_ = R_T_−R_1_.

Terminal compliance was estimated following Reymond et al. [22], where the terminal compliance of each terminal vessel, C_T,*i*_, was assumed to be proportional to the area compliance, C_A,*i*_, at the distal end of the terminal vessels:

$$C_{T,i}≅C_{T}\frac{C_{A,i}}{\sum C_{A,i}}$$ (8)

with C_T_ = ∑C_T,*i*_ the part of the total volume compliance attributed to peripheral vessels not included in the arterial tree model, assumed to be in the order of 20% of the total systemic vascular compliance. The total systemic vascular compliance is the sum of the volume compliance of all vessels and compliance of the terminal beds, so that $C_{V}=\sum_{n}^{i}C_{V,i}+\sum_{m}^{i}C_{T,i}$, where n = 117 is the total number of arterial segments and m = 62 is the number of terminal segments. To obtain volume compliance of each segment, the area compliance given by Eq (3) is integrated over the segment length. All values of terminal resistance and compliance can be found in S1 Table. Continuity of pressure and flow was imposed throughout the arterial network at bifurcations. Forward wave reflections were minimized by adapting the characteristic impedance of the tributaries so that the absolute value of the reflection coefficient was < 0.2 at all bifurcations. The cross-sectional area of the vessels was therefore slightly adjusted resulting in tapered-structure segments. Cross-sectional areas were determined by minimizing the reflection coefficient, subject to three conditions: (i) measured area is the average between the input and output areas (A_in_ and A_out_ respectively); (ii) A_in_ of tributaries is lower or equal to A_out_ of the parent, and (iii) for each segment A_out_ ≤ A_in_. Initial searching points were obtained by assuming linear tapering. The forward wave reflection coefficient is calculated as:

$$\Gamma =\frac{Z_{\mathrm{parent}}^{-1}-\sum Z_{\mathrm{daughter}}^{-1}}{Z_{\mathrm{parent}}^{-1}+\sum Z_{\mathrm{daughter}}^{-1}}$$ (9)

where Z is the characteristic impedance of the parent and daughter vessels.

#### Heart model

A model of the left ventricle (LV) was used at the proximal end of the arterial tree (root of the ascending aorta), simulating the blood flow pumped out of the LV. The LV model is based on the time-varying elastance model, which describes the variation of LV pressure (P_LV_) and volume (V_LV_) during a cardiac cycle.

$$E(t)=\frac{P_{\mathrm{LV}}(t)}{V_{\mathrm{LV}}(t)-V_{0}}$$ (10)

The interaction between the ventricle and the arterial tree is mainly produced during the ejection phase, when the aortic valve is open. To improve the simulation of the wave reflection phenomena that occur during this period between the ventricle and the aorta, an internal resistance of the LV was introduced. Taking this into consideration, the varying elastance model originally suggested by Sagawa [25] was adapted by Reymond et al. [22], leading to the following expression for the varying elastance of an ejecting heart:

$$E(t)=E^{*}(t)[1-\kappa Q(t)]$$ (11)

where E^*^ is the elastance that would be measured during a nonejecting isovolumic contraction, and κ a constant relating the internal resistance of the LV to the ventricular pressure during the same cardiac phase. Assuming that the elastance curve (when normalized with respect to its peak value) is similar in shape for all mammals, a normalized isovolumic elastance, E^*^, can be derived from Eq (11) using the global normalized elastance curves, E, reported by Senzaki et al. [26]. The constant κ was derived iteratively by minimizing the difference between the elastance curve resultant from the 1D model and the original elastance curve reported by Senzaki et al. [26]. The value of κ obtained was 55E-06 s/ml. Due to the lack of detailed horse data in current literature, assumptions needed to be made to set most of the input parameters necessary for the heart model. The value of end-diastolic pressure was taken as, P_end-dias_ = 16 mmHg, according to the value reported by Brown and Holmes [27] for a normal horse and well within the standard range (12–24 mmHg) reported in the literature for horses [28], whereas the end-systolic pressure was 113 mmHg [27]. The value of the dead volume of the LV was set to V_0_ = 0 ml. Initial reference values for stroke volume (SV = 900 ml/min) and ejection fraction (EF = 60%) were used to estimate the end-diastolic volume (EDV) and the end-systolic volume (ESV), wherewith initial values of minimal and maximal elastance were derived considering Eq (10). These values were further tuned to obtain a close match between the simulated aortic flow velocity and the aortic flow velocity measured from ultrasound imaging, which resulted in final values of E_min_ = 0.01 mmHg/ml and E_max_ = 0.26 mmHg/ml. The standard heart rate was set to 40 bpm, a normal physiological value for the horse at rest, whereas systolic duration was set to 478 ms [29].The heart model simulates the four main phases of the cardiac cycle, starting the loop at the onset of the isovolumic contraction phase, where the volume in the LV equals EDV (derived in the simulation from Eq (10), for a LV pressure equal P_end-dias_). With the contraction, the pressure in the ventricle rises over the aortic pressure, which causes the opening of the aortic valve and the start of the ejection phase. During this period, the ventricle-arterial interaction is described by the combination of Eqs (10) and (11). When the flow becomes negative, end systole is reached (aortic valve closes) and the relaxation phase takes place. The filling phase is set when the pressure in the ventricle drops below the initially assumed P_end-dias_, and the filling flow is modelled from the internal resistance of the LV (assumed as 0.003 mmHg·s·ml^-1^). The solution of the heart model is periodic; at the start of every cardiac cycle, EDV takes the value derived from the previous cardiac cycle.

#### Coronary model

Coronary arteries were modelled following Reymond et al. [22], assuming that changes in compliance, distensibility and resistance are proportional to the local time varying elastance of each vessel. For the right coronary, it was additionally assumed that the effect of the right ventricular contraction is smaller by a factor proportional to the ratio of maximal pressure in the two ventricles (PLV,max/PRV,max ≈ 3 [28]).

### Hemodynamic and wave reflection analysis using wave power

Wave power analysis (WPA) [30] was applied in different locations to study the dynamics of the waves. The method defines the wave power, *dπ*, as the product of the changes in pressure (*dP*) and flow (*dQ*), and equals the energy carried by the wave which is conserved at junctions. Wave power can be separated into its forward and backward components (subscripts (+) and (-), respectively), *dπ* = *dπ*_+_+ *dπ*_−_, where *dπ*_+_ = 1/(4∙*Z*_*c*_)(*dP*+*Z*_*c*_*dQ*)^2^ and *dπ*_−_ = −1/(4∙*Z*_*c*_)(*dP*−*Z*_*c*_*dQ*)^2^. The concept of wave power is analogous to wave intensity analysis [31], with *dπ*>0 indicating dominant forward waves and *dπ*<0 indicating dominant backward waves. The distance travelled by the waves to their reflection points can be estimated as the product of the transit time between a wave and its reflection and the local theoretical PWV. Since the wave travels twice the same distance, the final distance can be computed as *L* = (Δ*t*∙*PWV*_*theor*_)/2.

## Results

### General physiological parameters

Running the model without including gravity revealed a cardiac output of 33 L/min, an ejection fraction of 65% and a stroke volume 820 ml. Systolic/diastolic pressure in the aortic root was 114/70 mmHg, with a pulse pressure of 44 mmHg and a mean arterial pressure of 93 mmHg. Taking gravity into account, cardiac output was reduced to 30 L/min, with an ejection fraction of 59% and a stroke volume of 740 ml, whereas systolic/diastolic pressure increased to 131/88 mmHg, resulting in an almost unaltered pulse pressure of 43 mmHg and an increased mean arterial pressure of 111 mmHg. The distribution of cardiac output derived from the model for both configurations is summarized in Table 2. Table 3 shows the values of the Womersley number, maximum shear stress, mean values of the convective acceleration approximation and the Reynolds number, derived from the model at different locations in the arterial network, including and neglecting gravity. for both configurations of the model. On the other hand, the total vascular resistance resulted in a value of 0.17 mmHg*s/ml for the model without gravity, and 0.22 mmHg*s/ml for the model with gravity. Note that these values differ from the total combination of resistances in parallel, since the total vascular resistance also account for the resistances in series in the vessels.

**Table 2:** Distribution of cardiac output (CO) in the model.

Body parts	Model without gravity CO distribution (%).	Model with gravity CO distribution (%).	Reference [24] CO distribution (%).
**Heart**	4.2	5.4	5
**Brain**	15.4	10.5	10
**Muscle**	7.7	11.3	15
**Kidney**	18.2	20.7	20
**Splanchnic**	28.3	32.6	30
**Other**	26.2	19.5	20

**Table 3:** Womersley number, maximum shear stress, mean convective acceleration and Reynolds numbers, derived from the model with and without gravity at different locations along the equine arterial tree.

Artery		Prox Ao	Dist Ao	CCA	MA	EIA
**Womersley number *α***		35.47	15.05	6.02	3.78	8.02
**Maximum shear stress (*τ***_**max**_ **in Pa)**	**With gravity**	2.72	2.89	2.28	1.74	1.50
	**Without gravity**	3.05	3.11	2.09	1.79	1.70
**Mean convective acceleration ($\frac{\partial }{\partial x}(\int_{A}u^{2}dA)$ 10**^**−5**^ **in m**^**3**^**/s**^**2**^**)**	**With gravity**	57.32	-27.75	-0.12	0.27	-1.02
	**Without gravity**	68.67	-31.55	6.79	0.06	-1.32
**Reynolds number**	**With gravity**	2404	870	1160	98	278
	**Without gravity**	2660	749	2312	68	195

Prox Ao: proximal aorta; Dist Ao: distal aorta; CCA: common carotid artery; MA: median artery; EIA: external iliac artery

### Effects of gravity

**Fig 2:**
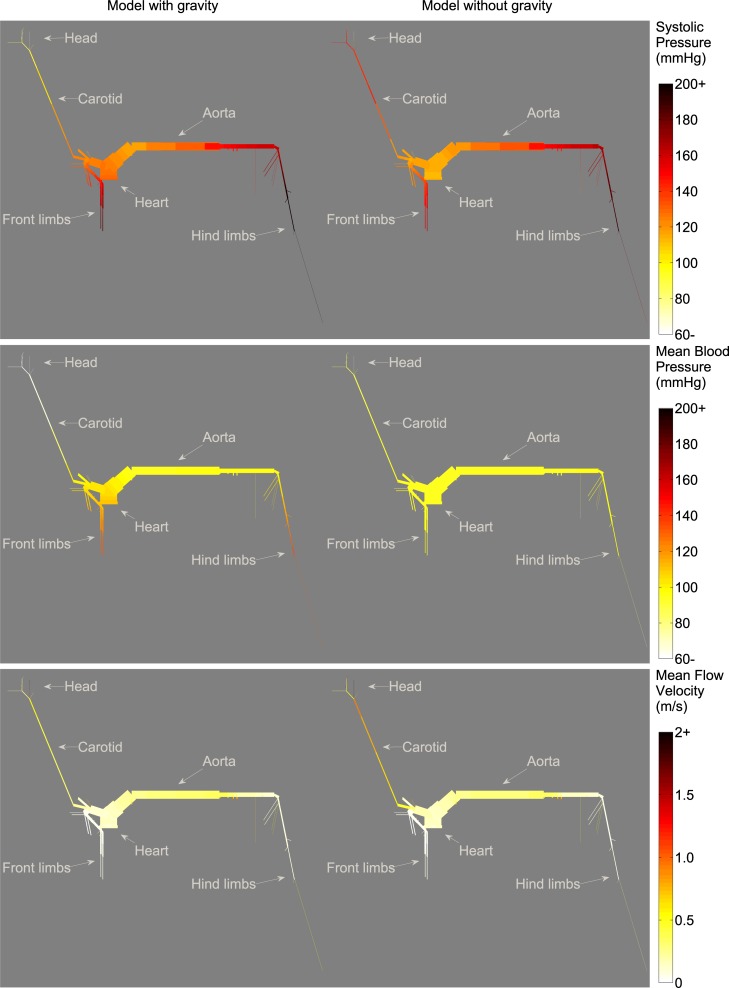
Distribution of systolic pressure, mean blood pressure and mean flow velocity over the complete arterial tree, comparing the model including gravity with the model neglecting gravity. Lower and higher values are indicated with colours varying from light to dark tones, respectively.

**Fig 3:**
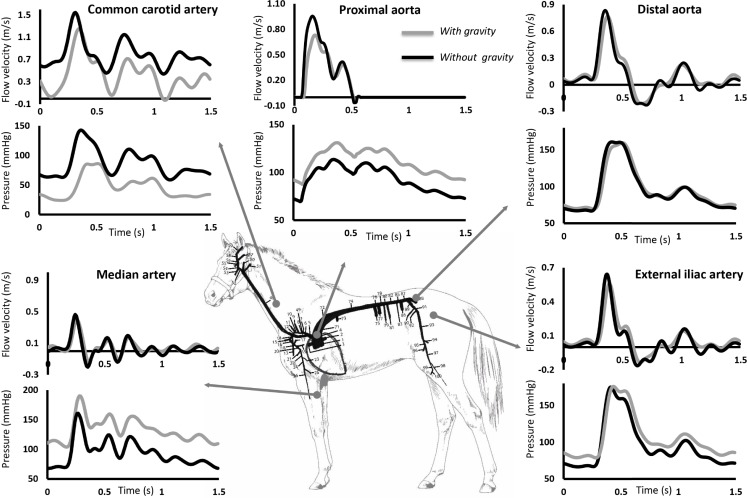
Model results (with and without gravity) of pressure and flow waveforms at various arterial locations: Common carotid artery, proximal aorta, distal aorta, median artery and external iliac artery.

To assess the importance of considering gravity in the model, the distribution of pressure and flow velocity was plotted all over the arterial tree, both with and without taking gravity into account (Fig 2). Considerable differences in pressure were most evident in the limb arteries, carotid arteries and the arteries of the head. Because of pressure amplification, systolic pressures were relatively high in the front and hind legs (>160 mmHg) when gravity was neglected while mean pressures were almost unaltered over the whole arterial tree. Considering gravity, systolic pressures in the front legs became even higher, whereas systolic pressures in the arteries of the head became low (<100 mmHg) and mean pressures increased from the head to the legs. Mean flow velocity distribution was similar for both configurations, with higher values for vessels in the splanchnic region and toward the head. A more direct comparison on the features of waveforms is displayed in Fig 3, with evident discrepancies in flow waveforms of the common carotid artery and in pressure waveforms for the proximal aorta, common carotid artery and median artery.

### Model predictions vs. *in vivo* measurements

Table 4 presents peak blood flow velocities and pressure data estimated from the 1D model in both situations, including and neglecting gravity, along with available *in vivo* measurements. Of note is the higher pulse pressure predicted by both models, in combination with the amplification of pulse pressure towards the periphery. Moreover, both models reveal an amplification in pressure along the aorta. An increase in systolic pressure from the ascending aorta to the iliac artery of ~54% was found for the model neglecting gravity, while it was reduced to ~34% when gravitational forces are included.

**Table 4:** Pressure and blood flow velocity predictions derived from the model with and without gravity and corresponding in vivo measurements at different locations along the equine arterial tree.

Artery		Prox Ao	Dist Ao	CCA	MA	EIA
**Segment number**		1	87	49	26	93
**Peak flow velocity (m/s)**	*In vivo*	0.742^*^	NA	0.846^*^	0.403^*^	0.457^*^
	Model without gravity	0.955	0.834	1.544	0.464	0.642
	Relative error (without gravity)^°^	29%	NA	82%	15%	40%
	Model with gravity	0.731	0.765	1.253	0.395	0.560
	Relative error (with gravity)^°^	2%	NA	48%	2%	23%
**Mean pressure (mmHg)**	*In vivo*	104.5^**^	119.7^**^	116.9^*^	NA	NA
	Model without gravity	92.82	94.63	88.61	93.54	94.77
	Relative error (without gravity)^°^	11%	21%	24%	NA	NA
	Model with gravity	110.74	95.74	64.04	132.34	105.54
	Relative error (with gravity)^°^	6%	20%	45%	NA	NA
**Systolic pressure (mmHg)**	*In vivo*	116.7^**^	134.4^**^	134.8^*^	NA	NA
	Model without gravity	113.62	160.76	145.08	160.68	174.93
	Relative error (without gravity)^°^	3%	20%	8%	NA	NA
	Model with gravity	131.11	159.78	103.44	190.17	175.73
	Relative error (with gravity)^°^	12%	19%	23%	NA	NA
**Diastolic pressure** **(mmHg)**	*In vivo*	92.2^**^	109.8^**^	101.9^**^	NA	NA
	Model without gravity	69.50	66.70	61.00	67.70	66.38
	Relative error (without gravity)^°^	25%	40%	40%	NA	NA
	Model with gravity	87.75	69.30	44.63	103.78	78.61
	Relative error (with gravity)^°^	5%	37%	56%	NA	NA
**Pulse Pressure (mmHg)**	*In vivo*	24.0^**^	24.2^**^	32.8^**^	NA	NA
	Model without gravity	44.12	94.06	84.08	92.97	108.55
	Relative error (without gravity)^°^	83%	288%	156%	NA	NA
	Model with gravity	43.36	90.47	58.81	86.39	97.13
	Relative error (with gravity)^°^	81%	274%	79%	NA	NA

Prox Ao: proximal aorta; Dist Ao: distal aorta; CCA: common carotid artery; MA: median artery; EIA: external iliac artery; NA: not applicable

*Measured in standing, non-sedated horses; mean of all investigated horses

**Measured in the anesthetised horse in dorsal recumbency; values of only 1 horse

°Relative error was calculated as ǀ (in vivo measured value–Modelled value) ǀ / in vivo measured value.

**Fig 4:**
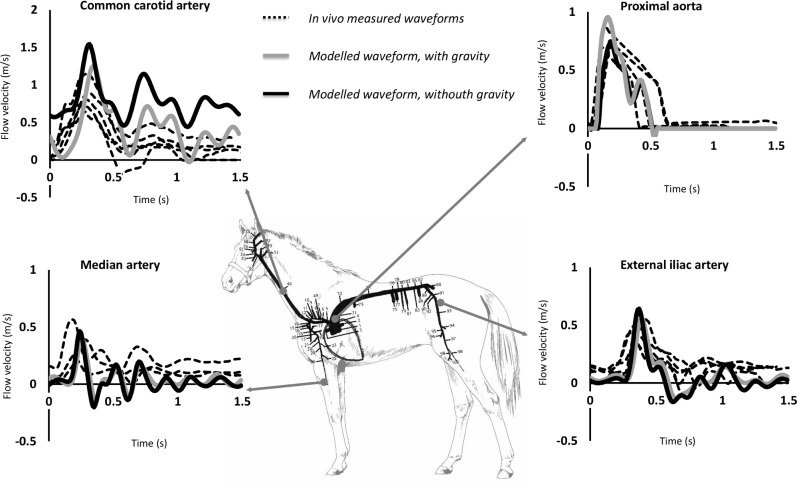
Model results (with and without gravity) compared with the averaged flow waveforms of all investigated horses at various arterial locations: Common carotid artery, ascending aorta, median artery and external iliac artery.

Fig 4 displays the modelled blood flow velocity waveforms, both with and without including gravitational forces, for the ascending aorta, common carotid artery and the main limb arteries (median artery and external iliac artery), compared with the measured waveforms in standing horses, derived using pulsed wave Doppler ultrasound. A relatively good similarity in waveform shape and their amplitude was found at all arterial locations, with relative errors for peak flow velocity between *in vivo* data, in the standing awake animal, and simulations without including gravitational forces of 29% for the ascending aorta, 82% for the common carotid artery, 15% for the external iliac artery and 40% for the median artery. When gravitational forces are included, relative errors improved to 2%, 48%, 2% and 23%, respectively.

**Fig 5:**
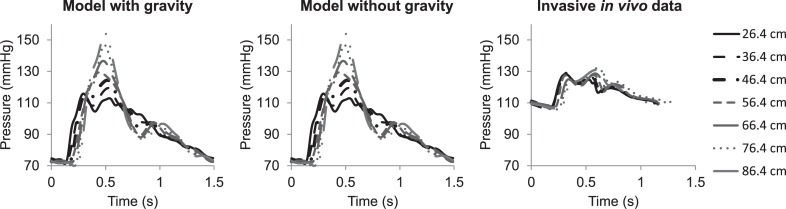
In vivo pressure waveforms compared with simulations, at seven locations along the aorta. Distances are expressed in centimetres distal from the aortic root.

Fig 5 compares *in vivo* invasively measured pressure waveforms along the thoracic and abdominal aorta (horse under general anaesthesia in dorsal recumbent position), with modelled pressure waveforms at the same locations (simulations resemble a non-anaesthetised horse), with and without considering gravitational forces. The effect of wave propagation is well captured by the model. Modelled mean, systolic and diastolic arterial pressures in the proximal aorta showed relative errors of 11%, 3% and 25%, respectively, compared to the invasively recorded pressure measurements (recorded under general anaesthesia with the horse in dorsal recumbent position). When gravitational forces are included relative errors changed to 6% for mean pressure, 12% for systolic pressure and 5% for diastolic pressure. For the distal aorta relative errors were slightly higher, 21% for mean pressure, 20% for systolic pressure and 40% for diastolic pressure. Including gravity had almost no influence on the derived pressures and thus the relative errors. Aortic PWV determined from the simulation was 5.3 m/s when gravity is neglected, changing to 5.2 m/s considering gravity, whereas the value obtained from *in vivo* data was 5.27 m/s (difference of ~0.6% when comparing with the model neglecting gravity, and ~-1.3% when comparing with the model including gravity).

### Wave power analysis (WPA)

**Fig 6:**
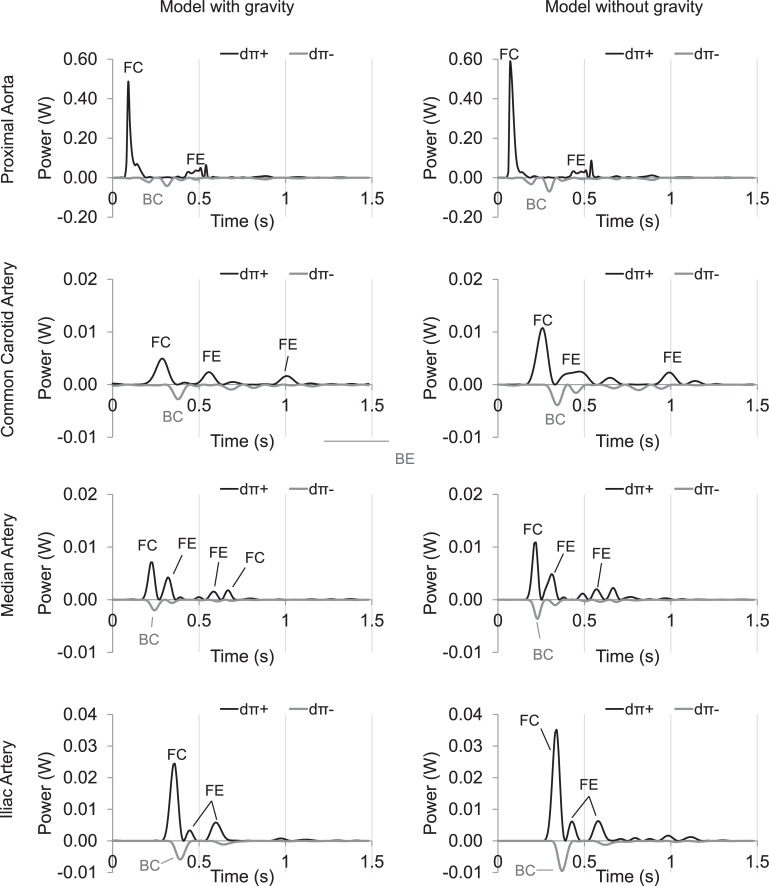
Wave power analysis at several locations of the arterial tree, comparing the model including gravity, with the model neglecting gravity. (FC: forward compression; FE: forward expansion; BC: backward compression; BE: backward expansion; 〖dπ〗_+ and 〖dπ〗_-: forward and backward components of wave power, respectively).

WPA is shown in Fig 6 for the proximal aorta, common carotid, median and external iliac artery. Models with and without including gravitational forces are compared. In both situations, the proximal aorta shows the three typical peaks: i) a forward compression (FC) wave generated by the systolic ejection, ii) a backward compression (BC) wave as the result of peripheral reflection, and iii) a forward expansion (FE) wave due to the slowing of ventricular contraction. Besides these normal peaks, a small FE can be visible at the different locations in mid-systole. The timing of occurrence of this wave coincides with the drop in flow signals for the median and iliac arteries. For all the considered locations, wave power was lower when gravitational forces were included. The largest difference between both situations is observed at the carotid artery, where for the configuration without gravity, a mid-systolic FE wave combines with the typical FE from the ventricular contraction, resulting in a wide FE wave; and this in a more drastic drop in the carotid systolic pressure of the model without gravity than the model with gravity (Fig 3).

## Discussion

Current literature provides only limited information on arterial hemodynamics in horses. In-depth fundamental research into equine intra-arterial pressures and flows is therefore expected to increase the understanding of aortic and arterial rupture in this species [14–17]. Today, due to technical limitations it is difficult to assess pressure and flow in the centrally located arteries and therefore peripheral measurements are usually extrapolated to the rest of the vascular network. The present study aimed to develop a 1D computer model for the equine arterial circulation comprising all major vessels of the arterial tree. The original 1D human model on which this equine model is based, has been validated and is used as a research tool in many studies [5, 32–35]. Recently, it formed the basis for the development of a 1D model in mice [36]. As aortic branching pattern of horses is completely different compared to humans, arterial segments needed to be redefined. *Ex vivo* measurements (diameter, length and branching angle) of each arterial segment along the equine arterial tree resulted in a unique dataset of the equine arterial anatomy (Table 1). Dimensions (lengths and diameters) of the equine arterial tree are much bigger than those of humans. Moreover, equine heart rate is lower at rest (28–45 bpm in horses and 60–100 bpm in humans) and the heart mass is much larger compared to humans (>1% of the body mass in horses and ~0.5% of the body mass in humans [24]). Both elements imply a much higher cardiac output in horses (~35 L/min compared with ~7 L/min in humans). Of note is the higher Womersley number in the horse, implying a considerably higher impact of inertia on the flow velocity profile, leading to flatter velocity profiles with steeper velocity gradients near the wall than those predicted by Poiseuille flow. As this leads to an improved estimation of viscous friction, it was important to include the Witzig-Womersley correction factor in the momentum equation.

### Importance of gravity

Existing 1D models usually ignore the effects of gravity, because most physiological measurements in humans are performed in supine position, with the heart being at the same level as the rest of the arterial tree. In horses most blood flow velocity and pressure measurements are performed on standing patients, and therefore the effect of gravity was incorporated in the present model. The gradient of pressure in the arterio-venous system, which causes flow, does not fluctuate considerably for the body in the supine position compared with that in the standing position; thus overall flow is similar in both situations. The transmural pressures on the contrary, are strongly different. Given the nonlinear pressure dependency of arterial stiffness, gravity was expected to exert an effect on wave propagation and wave morphology. This was indeed confirmed by our results.

In order to assess the impact of gravitation, two configurations of the model (with and without gravity) were compared (Figs 2–6). Taking into account gravity generally improved predicted peak flows. Relative errors improved from 15–82% for the model without gravity to 2–48% for the model including gravity. While peak flow predictions improved when including gravity into the model, relative error for mean pressure, diastolic pressure and systolic pressure did not improve when gravity was included, with relative errors ranging from 3–40% without gravity and 5–56% with gravity included. Fig 2 shows that, when gravity is neglected, mean blood pressure is practically the same in all conduit arteries. As was expected, including gravity into the model revealed more variability in mean pressure throughout the arterial tree, with lower values toward the head and higher values toward the limbs. Fig 5 clearly shows the similarities in pressure waveforms of the descending aorta with and without gravity. These similarities in pressure waveforms are due to the assumption of a horizontal motion of blood (angle = 0) in the descending aorta, causing no influence of gravity.

### Wave power analysis

As changes in arterial pressure waves are associated with alterations in the contour of the arterial flow profiles [37], the pronounced oscillations during flow wave measurements at different arteries in horses probably indicate pressure waves returning from the periphery at multiple reflection sites, starting during systole and continuing during diastole. Wave power analysis performed at several locations revealed important wave reflections mainly during systole. Wave power patterns were most complex for the common carotid artery, which also displayed the biggest delay between the first FC wave and its peripheral reflection (BC wave) coming from the head. This was somewhat expected considering that the wave has to travel forth and back along the carotid artery. The reflection of the first forward peak at the peripheral site in the head was higher when gravity was included in the model (~56% with gravity vs. ~36% without gravity). The presence of a mid-systolic forward expansion wave immediately after the first peak generated by the heart ejection (Fig 6), was responsible for the drop of pressure and flow velocity that resulted in narrow systolic peaks in pressure and flow waveforms of the investigated vessels. In order to understand the origin of this suction wave, we estimated the distance travelled by the wave to the site of re-reflection. By combining the time difference measured from the foot of the waves with the local theoretical PWV in the involved segments, very short distances were derived. These re-reflections might be occurring locally in the network rather than at the heart level, and may be related to a mismatch of junctions in the peripheral branches. Higher efforts to minimize forward wave reflections at these sites are needed.

### Limitations and future work

In general flow waveform patterns (morphology) are well captured by the model, especially by the one including gravity while discrepancies in amplitude are quite obvious (Fig 4), this is probably due to several limitations of this model, which will be explained clearly in this section.

Since the present model is based on averaged data, it enables us to predict generic local pressures and flow profiles in all investigated arterial segments. A fully quantitative validation, however, would require a detailed horse-specific approach, tuning of all input parameters that define the model to each specific animal (such as geometry, elastic properties, peripheral resistance, and cardiac parameters) and comparing the outcomes of the model with *in vivo* measurements in that specific animal. Such an approach is technically challenging and almost impossible. Therefore, even if we consider the present model to be representative for the average healthy horse, waveforms should only be compared qualitatively and not quantitatively to individual measurements.

The lack of literature data on equine hemodynamics was the major challenge to develop an equine 1-D model. Tuning the model parameters was based on plausible assumptions and scaling factors between human and equine patients. Further fine-tuning of the input parameters to equine physiology will be necessary to obtain a closer match with *in vivo* flow profiles. Branching pattern and dimensions of the arterial tree, both largely defining flow wave patterns, are well integrated in this model. However, branching patterns and arterial dimensions can vary significantly between individual horses. Other important parameters, influencing flow velocity and pressure waves morphology are arterial elasticity and peripheral resistance. Both, arterial elasticity and peripheral resistance largely define diastolic flow [38] and pulse pressure [33], two parameters still showing large differences between measured values and modelled ones, with relative errors for diastolic pressure ranging from 5–60% and for pulse pressure ranging from 81 to 288%. Equine arterial elasticity and peripheral vascular resistance are therefore interesting criteria to further unravel in the future. In addition, in our simulations the compliance distribution and peripheral resistances remain the same for both, the model with gravity and the model without gravity. Neglecting the autoregulation mechanisms that lead to a cardiovascular response to control blood flow and pressure levels during postural changes, can also partially explain the differences found in our simulations and *in vivo* data.

The limited research possibilities in equines form another restrictive factor in the development of the equine 1D model. Due to ethical concerns, invasive aortic blood pressure was only collected in one horse, which is a limitation of this study. Moreover these aortic blood pressures were collected in anaesthetized, dorsally recumbent animals, while ultrasound and pressure measurements at the carotid artery were performed in non-sedated, standing horses. This complicates comparison between *in vivo* measurements and modelled findings. The present model does not yet account for the changes in physiological parameters due to anaesthesia or dorsal recumbent position. Anaesthesia tends to slow down heart rate, reduce both cardiac output and pulse pressure and likely leads to modulations in resistance of vascular beds and mechanical properties of arteries. Changes in smooth muscular tone modulate distensibility and stiffness. The recumbent position of the horse has a huge effect on intrathoracic and arterial transmural pressure, modulating stiffness and leading to important volume shifts of blood affecting cardiac filling and preload and, via the Frank-Starling mechanism, cardiac contractility. In addition, the model does not include physiological control mechanisms such as the baroreflex that neurologically modulates cardiovascular function via sympathetic/parasympathetic mechanisms. When a body changes from the supine to the standing position, these baroreceptor control mechanisms are activated to limit mean arterial pressure decrease to only a few mmHg. The restoring mechanisms include an increase in cardiac output (mainly an increase in heart rate), as well as an increase in systemic vascular resistance.

Next to this, discrepancies in pressure and flow waveforms between measured and obtained modelled values may be affected by angle correction. In order to obtain standardised images, angle correction was set at 45° when collecting flow velocity profiles in the standing horse. Measurements were optimized for alignment with the flow but this alignment was not always perfect and flow was probably not always captured in the centre of the artery, which implies that captured flow velocities are only an approximation of the true flow profiles.

Discrepancies in pressure and flow waveforms may also be affected by the exclusion of the entire systemic and pulmonary circulation, making the model an open loop system that requires boundary conditions: a cardiac time-varying elastance model at the inlet, and the Windkessel model at the terminal ends. Moreover, the cerebral arterial tree is not included in detail in the equine model, only a simplified representation containing the major vessels that supply the cerebral circulation. A more detailed description of the cerebral arterial tree may provide better predictions of pressure and flow waveforms in the carotid artery and smaller vessels of the head circulation, as has been previously shown for the human and murine models [39]. Furthermore, in the present design, the model used for coronary arteries is simplistic. Last but not least, development of the present model was based on data obtained on a limited number of horses, without accounting for cardiovascular variations due to age or gender.

Nevertheless, despite individual differences in absolute values of flow velocities and arterial pressures, flow wave morphology is well captured by the model as shown in Fig 4. Fig 5 evidences the effect of wave propagation along the aorta both in the measured and the modelled pressure profiles. This indicates the added value of the model for studying trends in arterial flow dynamics in horse populations.

In the future this computer model may be useful to predict changes in flow profiles and local pressures under specific circumstances or conditions (age, exercise). During high-intensive exercise, heart rate may rise up to 8 times above the resting rate and total aerobic capacity can reach a 40-fold increase, which is much greater compared to human athletes. By altering input parameters of the horse-specific model, this model might predict local pressures and flow profiles during these extreme circumstances and contribute to the understanding of the relatively high incidence of sudden death during exercise due to arterial rupture [14–17]. As increasing age increases the risk of arterial disorders (arterial rupture during exercise, during parturition or after phenylephrine administration) [14, 18–20], it might also be interesting to use the present model to study the effect of age on arterial hemodynamics. Moreover the development and adjustment of this kind of computer models, could lead to a better understanding of some intensively studied, but poorly understood clinical situations such as exercise induced pulmonary hemorrhage.

## Conclusions

A 1D computer model for the equine arterial circulation has been developed and this provided a unique anatomical dataset for horses. *Ex vivo* anatomical measurements were combined both with literature data and physiological information from ultrasound analysis in order to predict pressure and flow waveforms in the equine arterial tree by means of 1D modelling. The qualitative validation of the model was carried out by comparing the results with average flow velocities and pressures measured *in vivo* in horses. Despite its generic character and limitations, outcomes from the model showed plausible predictions of pressure and flow waveforms throughout the considered arterial tree. Simulated flow waveforms reproduce important features observed in ultrasound Doppler images, especially the oscillating pattern (most pronounced at the external iliac artery, median artery and common carotid artery). Adapting the model by taking into account gravity further improved predicted waveforms. Thanks to wave power analysis, the contours of the arterial flow profiles could be explained. Despite the shortcomings of *in vivo* measured pressures (aortic pressures measured under general anaesthesia with the horse in dorsal recumbent position), modelled pressure data seem in line with invasive measurements. We believe that the present model may be useful, not only to explain flow wave patterns in horses, but also to predict changes in flow profiles and local pressures as a result of strenuous exercise or altered arterial wall properties related to age, breed or gender.

## Supporting information

S1 Table Terminal impedance data. (DOCX)
