# Correction: A 1D computer model of the arterial circulation in horses: An important resource for studying global interactions between heart and vessels under normal and pathological conditions

**DOI:** 10.1371/journal.pone.0225396

**Published:** 2019-11-13

**Authors:** Lisse Vera, Daimé Campos Arias, Sofie Muylle, Nikos Stergiopulos, Patrick Segers, Gunther van Loon

The arrows in Fig 3 are not aligned with the correct arteries. Please see the complete, correct Fig 3 here.

**Fig 3:**
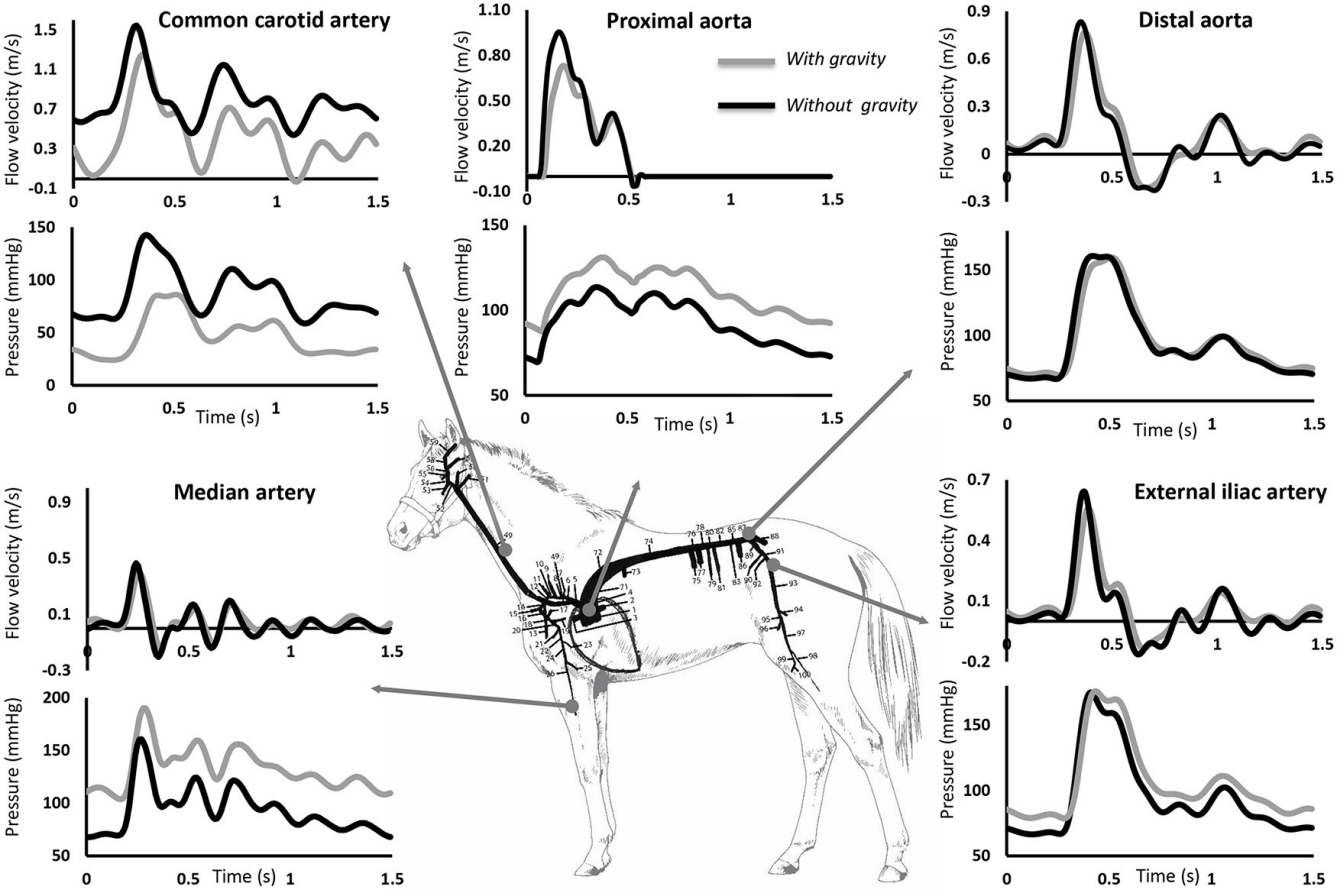
Model results (with and without gravity) of pressure and flow waveforms at various arterial locations: Common carotid artery, proximal aorta, distal aorta, median artery and external iliac artery.
