# Supplementary material for: A 1D computer model of the arterial circulation in horses: An important resource for studying global interactions between heart and vessels under normal and pathological conditions
Source: PLoS One. 2019 Aug 21;14(8):e0221425. doi: 10.1371/journal.pone.0221425 (PMC6703698; doi:10.1371/journal.pone.0221425)
Supplement: S1 Table — (DOCX) [file pone.0221425.s001.docx]

**S1 Table. Terminal impedance data.**

| **Arterial Segment Number** | **Terminal resistance mmHg*s/ml** | **Terminal compliance**  **10^-2^ ml/mmHg** |
| --- | --- | --- |
| **2** | 7.22 | 14.36 |
| **3** | 7.22 | 14.36 |
| **7** | 32.80 | 7.97 |
| **9** | 29.82 | 0.05 |
| **11** | 10.98 | 7.53 |
| **13** | 32.80 | 11.01 |
| **15** | 55.44 | 1.34 |
| **17** | 55.44 | 0.61 |
| **19** | 55.44 | 25.80 |
| **21** | 55.44 | 0.33 |
| **23** | 55.44 | 6.34 |
| **25** | 67.61 | 1.31 |
| **26** | 64.46 | 1.65 |
| **28** | 32.80 | 3.62 |
| **30** | 29.82 | 0.05 |
| **32** | 10.98 | 3.98 |
| **35** | 55.44 | 11.00 |
| **36** | 55.44 | 3.78 |
| **38** | 55.44 | 1.46 |
| **40** | 55.44 | 25.80 |
| **42** | 55.44 | 0.33 |
| **44** | 61.60 | 7.55 |
| **46** | 67.61 | 1.31 |
| **47** | 64.46 | 1.65 |
| **50** | 1.10 | 0.48 |
| **51** | 8.55 | 0.71 |
| **53** | 8.55 | 1.50 |
| **55** | 8.62 | 0.04 |
| **57** | 8.92 | 0.10 |
| **58b** | 9.00 | 1.19 |
| **59** | 9.08 | 0.20 |
| **61** | 1.10 | 0.48 |
| **62** | 8.55 | 0.71 |
| **64** | 8.55 | 1.50 |
| **66** | 8.62 | 0.04 |
| **68** | 8.92 | 0.10 |
| **70** | 9.08 | 0.20 |
| **73** | 3.55 | 2.78 |
| **75** | 1.96 | 7.34 |
| **77** | 3.03 | 4.04 |
| **79** | 1.80 | 3.62 |
| **81** | 1.80 | 3.63 |
| **83** | 22.55 | 0.24 |
| **84** | 22.55 | 0.24 |
| **86** | 3.51 | 1.22 |
| **88** | 5.52 | 21.68 |
| **90** | 47.31 | 0.66 |
| **92** | 23.13 | 0.12 |
| **94** | 47.31 | 2.97 |
| **96** | 47.31 | 0.65 |
| **79b** | 9.00 | 1.20 |
| **98** | 47.31 | 0.07 |
| **99b** | 57.70 | 6.21 |
| **100** | 57.70 | 0.47 |
| **101** | 5.52 | 21.68 |
| **103** | 23.13 | 0.12 |
| **105** | 55.02 | 0.70 |
| **107** | 47.31 | 2.97 |
| **109** | 47.31 | 0.65 |
| **111** | 47.31 | 0.07 |
| **112b** | 57.70 | 6.21 |
| **113** | 52.57 | 0.47 |
